# The Higher-Order Prover Leo-II

**DOI:** 10.1007/s10817-015-9348-y

**Published:** 2015-09-22

**Authors:** Christoph Benzmüller, Nik Sultana, Lawrence C. Paulson, Frank Theiß

**Affiliations:** 1 Department of Mathematics and Computer Science, Freie Universität Berlin, Berlin, Germany; 2 Cambridge University Computer Lab, Cambridge, UK; 3 Saarland University and Bold Solutions, Saarbrücken, Germany

**Keywords:** Automated theorem proving, Higher-order logic, Proof assistant

## Abstract

Leo-II is an automated theorem prover for classical higher-order logic. The prover has pioneered cooperative higher-order–first-order proof automation, it has influenced the development of the TPTP THF infrastructure for higher-order logic, and it has been applied in a wide array of problems. Leo-II may also be called in proof assistants as an external aid tool to save user effort. For this it is crucial that Leo-II returns proof information in a standardised syntax, so that these proofs can eventually be transformed and verified within proof assistants. Recent progress in this direction is reported for the Isabelle/HOL system.

## Motivation and Background

Leo-II is a standalone, resolution-based higher-order (HO) automated theorem prover (ATP) that is designed for cooperation with specialist provers for fragments of HO logic. The idea is to combine the strengths of the different systems. On the other hand, Leo-II itself, as an external reasoner, aims to support HO proof assistants such as Isabelle/HOL [47], HOL [34] or HOL Light [36].

The predecessor of Leo-II, Leo-I [16], was originally designed as a fully-automated subsystem of the interactive proof assistant and proof planner $\varOmega$ mega [53]. Similar in spirit to Andrews’ pioneering TPS system [4], Leo-I was intended to solve selected subgoals automatically in order to save user interaction or support a proof planner. Technically, however, the resolution-based Leo provers differ significantly from the matings-based TPS system. Leo-I was hard-wired to the $\varOmega$ mega proof assistant. The prover already supported native (versus Huet’s axiomatic) treatment of the extensionality principles [8] and it cooperated with first-order (FO) ATPs via the flexible $\varOmega$ Ants agent architecture within $\varOmega$ mega [26]. Both native extensionality treatment and cooperation with specialist reasoners for fragments of HO logic have been adopted in Leo-II, and also in other systems, most notably in the recent Satallax prover by Brown [30].

Leo-II’s calculus is based on Resolution by Unification and Equality [33]. That is, unification constraints are disagreement pairs, and are amenable to resolution. The prover supports primitive equality handling (in Leo-I equality was expanded using Leibniz’ definition), calculus-level treatment of choice, and depth-bounded HO pre-unification.

The rest of the article is structured as follows. More information on the theory and background of Leo-II is provided in Sect. 2. The prover’s main loop and its direct collaboration with FO ATPs are outlined in Sect. 3. An example proof of Leo-II is presented in Sect. 4. The prover can also be used in interactive mode; however, this feature is not described here. Leo-II also implements term sharing and term indexing (Sect. 5). Leo-II’s native input language is TPTP THF0 [64]. Section 6 describes how the development of the THF0 language, which in turn fostered significant improvements in HO theorem proving, has been paralleled and influenced by the development of Leo-II. In that section it is also explained why Leo-II (and other THF0 compliant provers) can readily be used for automating a wide spectrum of quantified non-classical logics via semantic embeddings. Proof certificates, which have been a central objective of the Leo provers from the beginning, are covered in Sect. 7. Leo-II’s proof certificates are exploited in the prover’s recent integration with Isabelle/HOL, through which Leo-II proofs can now be transformed and verified (Sect. 8). Section 9 summarises selected applications of Leo-II and points to integrations of Leo-II with other systems.

The Leo-II prover can be easily deployed and installed. The source code is freely available from http://www.leoprover.org under a BSD-style license.

## Foundation of Leo-II

ATPs based on the resolution principle, such as Vampire [51], E [52], and SPASS [66], have reached a high degree of sophistication. They can often find long proofs even for problems having thousands of axioms. However, they are limited to FO logic. HO logic extends FO logic with lambda notation for functions, and with function and predicate variables. It supports reasoning in set theory, using the obvious representation of sets by predicates. HO logic is a natural language for expressing mathematics, and it has also found much use in formal verification. Moving from FO to HO logic requires a more complicated proof calculus, but it often allows much simpler problem statements. HO logic’s built-in support for functions, predicates and sets (as characteristic functions) often leads to shorter proofs. Moreover, elementary identities (such as the distributive law for union and intersection) turn into difficult problems when expressed in FO form.^1^

Benzmüller et al. [25] give a tour of models for HO logic. A family of weak models for HO logic is presented, for which complete calculi can be defined. In a sense, equality is ‘native’ in HO logic—for instance, the weakest of these models validates $\beta$-equivalence. The strongest of these weak systems is called *Henkin semantics*, and it is the semantics under which Leo-II works.

Unlike in FO logic, terms in HO logic have a native equality defined on them through $\lambda$-conversion. In Henkin semantics, this relation corresponds generally to $\alpha \beta \eta$-conversion. In HO logic, terms may be function-valued, and formulas are simply Boolean-valued terms. Term equivalence is taken to be modulo $\lambda$-conversion. Terms are represented, and $\beta \eta$-reduced, in Leo-II as graphs.

*Comprehension* is another strength of HO logic over FO logic. Comprehension is a device for defining sets through formulas. In FO logic, comprehension axioms need to be explicitly stated, but these axioms are native to HO logic since sets are defined *as* formulas.^2^ Benzmüller and Brown [15] identify comprehension as enabler for significantly shorter proofs in HO logic, compared with using FO logic.

Handling *equality* is more challenging in HO logic since it now applies to function-valued and Boolean-valued terms, and arriving at Henkin completeness requires handling the extensionality of functions and propositions. The respective axiom and scheme for *Boolean extensionality* (or *propositional extensionality*) and *functional extensionality* are $\forall X^o Y \cdot (X \longleftrightarrow Y) \longrightarrow X = Y$ and $\forall F^{\tau \rightarrow \sigma } G \cdot (\forall X^\tau \cdot F X = G X) \longrightarrow F = G$. As with equality-handling in FO logic, better performance is achieved by extending a proof calculus with equality-related rules rather than adding the characterising axioms to the logic [27]. The particular equality and extensionality rules of Leo-II have their roots in the work of Benzmüller [7].

Leo-II also provides a calculus-level treatment of the *axiom of choice* (AC). The solution in Leo-II [19] is inspired by work of Mints [44]. Choice is related to *Skolemization*. In HO logic, Skolemization is not as straightforward as in FO logic [43]. Naïve Skolemization is unsound wrt Henkin models that invalidate AC, and incomplete wrt Henkin models that validate AC [5] [14, Sect. 3.2].

Leo-II is a *resolution*-based prover. In FO resolution-based theorem proving, *clause normalisation* is only carried out once at the beginning of the process. In HO theorem proving, clause normalisation might be carried out several times (at different points during the proof process) since variables may be instantiated with formulas, and this may turn normal clauses into non-normal ones.

In FO logic, *unification* is decidable, and it is used as an eager filter during resolution. HO unification is undecidable in general, so it is used more carefully. Leo-II relies on a variant of Huet’s *pre-unification* procedure, which is semi-decidable. It works by accumulating flex–flex unification pairs as unification constraints (in flex–flex unification pairs both terms to be unified have variables at head position). When a clause consists only of flex–flex constraints then it is considered to be *empty*, since, as Huet showed [38], such a system of equations always has solutions. Thus, by employing unification constraints Leo-II delays and avoids unnecessary enumerations and applications of certain unifiers. In addition to this, Leo-II’s unification procedure interprets logical constants, such as conjunction, equality, etc.

Resolution and factorisation may be applied to the unification constraints too. Despite the theoretical benefit of lazy filtering, this produces problems in practice owing to accumulation of clauses, as described by Benzmüller [6, Sect. 3.3]. Though it was originally intended as an alternative option for Leo-II’s architecture, lazy unification has not yet been implemented. Eager unification in Leo-II works as follows: pre-unification is applied to clauses with a predefined depth bound (e.g. maximally five^3^ nestings of the branching flex-rigid rule; modulo this depth-bound HO pre-unification becomes decidable, but at the cost of incompleteness—also for Leo-II). The solved unification constraints are exhaustively applied in the resulting clauses, and any remaining flex–flex unification pairs are kept as unification constraints of the result clause. Pre-unification may return an empty clause—that is, a clause which is either literally empty or which consists only of flex–flex unification constraints, which always have a solution.

Unification is used to find instantiations of variables of arbitrary type. In HO automated theorem proving, an additional form of instantiation is required for completeness. This form of instantiation, which is called *primitive substitution*, only concerns predicate variables. For example, in order to prove $\exists P \cdot P$ or $\exists Q\exists X \cdot Q\,X$ we cannot use unification. Guessing instantiations for such variables is a comprehensive challenge since the search is infinitely-branching. Whereas in FO logic one can have a complete resolution calculus using only the factorisation and resolution rules, in HO resolution we need an additional rule for primitive substitution.

### Calculus

We sketch the rules of Leo-II’s extensional RUE calculus. More details are presented in earlier publications [13, 20, 58, 59].

#### Normalisation rules

These rules deal with the normalisation of clauses. They are straightforward, except for a special purpose, additional rule used for the exhaustive instantiation of some finite types $\tau$ having cardinality *n*. The rule instantiates *n* clauses, each with a different term of type $\tau$. Currently, this only applies when $\tau$ is *o*, $o\rightarrow o$ or $o\rightarrow o\rightarrow o$. For example, when applied to clause $\mathbf {C} \vee [\forall P_{o\rightarrow o} \cdot q_{(o\rightarrow o)\rightarrow o}\ P]^{\mathrm {t}\mathrm {t}}$ the special purpose normalisation rule introduces the clauses $\mathbf {C} \vee [q\ \lambda X_o \cdot X]^{\mathrm {t}\mathrm {t}}$, $\mathbf {C} \vee [q\ \lambda X_o \cdot \lnot X]^{\mathrm {t}\mathrm {t}}$, $\mathbf {C} \vee [q\ \lambda X_o \cdot \top ]^{\mathrm {t}\mathrm {t}}$ and $\mathbf {C} \vee [q\ \lambda X_o \cdot \bot ]^{\mathrm {t}\mathrm {t}}$ ($[\cdot ]^{\mathrm {t}\mathrm {t}}$ and $[\cdot ]^{\mathrm {f}\mathrm {f}}$ denote literals with positive and negative polarity).

#### Extensionality rules

To avoid the challenging extensionality axioms in the search space, Leo-II implements a native support for extensionality reasoning based on the following rules (where $X^\tau$ is a fresh variable and $\mathtt {sk}^\tau$ a Skolem term):
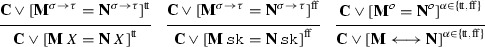


The rules operating on negative equality literals, i.e., unification constraints, are integrated with in Leo-II’s pre-unification procedure. The positive rules are combined with the normalisation rules.

#### Unification

This set of rules implements a variant of Huet’s pre-unification procedure that is augmented with the negative extensionality rules from above and which employs a search depth limit as parameter). The rules operate on unification constraints. The procedure, when applied to a given clause $\mathbf {D} \vee \mathbf {U}$, where $\mathbf {U}$ is set of unification constraints, returns a finite set of clauses of form $\sigma (\mathbf {D}) \vee \sigma (\mathbf {F}) \vee \sigma (\mathbf {B})$, where $\sigma$ is a substitution, $\mathbf {F}$ is a possibly empty set of flex–flex constraints, and $\mathbf {B}$ is a possibly empty set of non-normal literals obtained from applications of the Boolean extensionality rule.^4^ Subsequent normalisation of such clauses may be required.

#### Resolution, Factorisation and Primitive Substitution

The resolution and factorisation rules in Leo-II introduce unification constraints, which Leo-II attempts to (extensionally) pre-unify eagerly modulo the given unification depth, instead of permanently delaying them as in Huet’s constrained resolution approach [37].
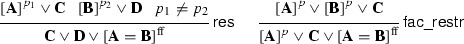


The primitive substitution rule, which is related to Huet’s splitting rule [39, 40] and Andrews’s primitive substitutions [2], guesses the top-level logical structure of the instantiation term $\mathbf {P}$, while further decisions on $\mathbf {P}$ are delayed. The hope is that they can eventually be determined by pre-unification in subsequent resolution steps. Generally, however, subsequent applications of primitive substitution rule are permitted and the deeper logical structure of $\mathbf {P}$ may thus be guessed later. It is an open challenge to suitably restrict this rule without threatening completeness.
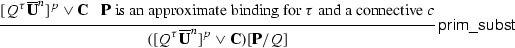


As an example consider the formula $\exists Q \exists X. \; Q\,X$. Negating and normalising the formula gives the clause $[Q^{\sigma \rightarrow o}\,X]^{\mathrm {f}\mathrm {f}}$. Rule prim_subst offers the clause $[\lnot H\,X]^{\mathrm {f}\mathrm {f}}$ by using $\lambda X \cdot \lnot H\,X$ as approximate binding for ${\sigma \rightarrow o}$ and $\lnot$. Further normalisation and resolution will yield a singleton clause consisting of a flex–flex constraint—that is, an effectively empty clause.

#### Choice

Recent versions of Leo-II also support a native treatment of choice. As for extensionality, the motivation is to avoid the choice axiom(s) in the search space. More details have been published elsewhere [13, 20].

## Cooperative Theorem Proving in Leo-II

Like many other provers, Leo-II spends its time looping during its exploration of the search space—executing its *main loop*. By *search space* we mean the totality of clauses surveyed by Leo-II during its execution. Each iteration of this loop might generate new clauses, thus contributing to the representation of the search space that is kept by Leo-II. Each iteration does *not* change the satisfiability of the problem and its search space; this is an invariant of a prover’s main loop.

Unlike many provers Leo-II keeps an additional representation of the search space. This is used to store the input to external provers. The contents of this store are produced by translating the clauses in the main store. The source clauses consist of HO clauses, and the target clauses are encoded in the target logic. Since Leo-II currently only cooperates with FO provers, the target clauses consist of FO clauses.

**Fig. 1:**
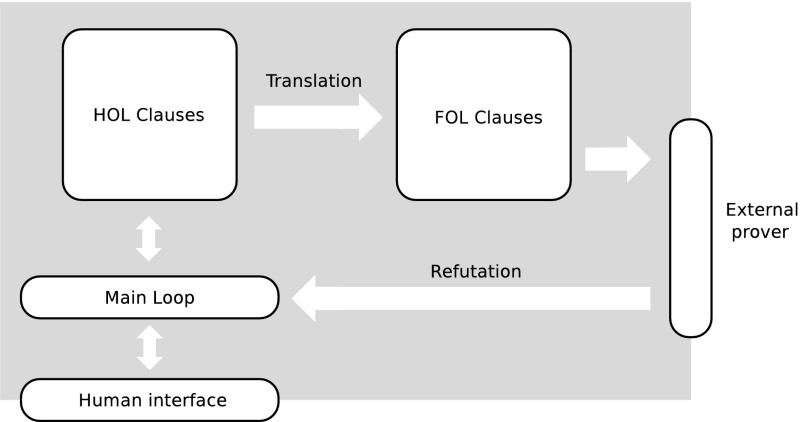
The main components involved in Leo-II’s cooperation with other provers

The FO clauses are accumulated during iterations of Leo-II’s main loop, and are periodically sent to the external prover with which Leo-II is cooperating. If the external prover finds the FO clauses to be inconsistent then, assuming that the translation was sound, it implies that the original HO logic clauses must also be inconsistent. This refutation is accepted by Leo-II, and presented to the user as a refutation of the initial conjecture. This setup is sketched in Fig. 1.

Various translations from HO logic to FO logic are implemented in Leo-II [19]. These translations differ in the amount of information they encode in the resulting FO formulas. Encoding less information can lead to incompleteness. Leo-II also implements a method devised by Claessen et al. [32], who describe an analysis on the cardinalities of types in order to safely erase some information. As part of this analysis, SAT problems are generated, and these are processed by MiniSat via an interface adapted from Satallax. The integration of more recent improvements of these methods [29] remains future work.

## Example Proof in Leo-II

We briefly illustrate Leo-II’s proof search for TPTP example SEV288ˆ5, which states that Leibniz equality is identical to primitive equality (in Henkin semantics):

$$\begin{aligned} (\lambda X_\alpha \lambda Y \forall Q \cdot \; Q X \longrightarrow Q Y) = (\lambda X \lambda Y \cdot \; X = Y) \end{aligned}$$

Initially, the prover negates the conjecture and expands any contained defined constant symbols. In our example, $\longrightarrow$ is defined as $\lambda A \lambda B \cdot \; \lnot A \vee B$. Because all terms in Leo-II are kept in $\beta \eta$-normal form, the following clause is obtained (where $[\cdot ]^{\mathrm {f}\mathrm {f}}$ denotes a literal with negative polarity):

$$\begin{aligned}{}[(\lambda X_\alpha \lambda Y\forall Q \cdot \; \lnot Q X \vee Q Y) = (\lambda X \lambda Y \cdot \; X = Y)]^{\mathrm {f}\mathrm {f}}\end{aligned}$$

Negated equation literals are treated by Leo-II as unification literals, to which the prover applies its extensional pre-unification algorithm. First, the outermost $\lambda$-abstractions are replaced, that is, functional extensionality is applied to obtain

$$\begin{aligned}{}[\forall X_\alpha \cdot \; (\lambda Y \forall Q \cdot \; \lnot Q X \vee Q Y) = (\lambda Y \cdot \; X = Y)]^{\mathrm {f}\mathrm {f}}\end{aligned}$$

Next, the leading quantifier is eliminated (*a* is a fresh Skolem constant)

$$\begin{aligned}{}[(\lambda Y \forall Q \cdot \; \lnot Q a \vee Q Y) = (\lambda Y \cdot \; a = Y)]^{\mathrm {f}\mathrm {f}}\end{aligned}$$

This procedure is repeated to obtain (*b* is a fresh Skolem constant)

$$\begin{aligned}{}[(\forall Q \cdot \; \lnot Q a \vee Q b) = (a = b)]^{\mathrm {f}\mathrm {f}}\end{aligned}$$

Syntactical pre-unification fails at this point, nevertheless Leo-II’s extended pre-unification process continues and applies Boolean extensionality to obtain

$$\begin{aligned}{}[(\forall Q \cdot \; \lnot Q a \vee Q b) \longleftrightarrow (a = b)]^{\mathrm {f}\mathrm {f}}\end{aligned}$$

This clause is subsequently normalised and the following clauses are obtained

$$\begin{aligned}{}[Q a]^{\mathrm {f}\mathrm {f}}\vee [Q b]^{\mathrm {t}\mathrm {t}}\vee [a = b]^{\mathrm {t}\mathrm {t}}\quad [q a]^{\mathrm {t}\mathrm {t}}\vee [a = b]^{\mathrm {f}\mathrm {f}}\quad [q b]^{\mathrm {f}\mathrm {f}}\vee [a = b]^{\mathrm {f}\mathrm {f}}\end{aligned}$$

Then, Leo-II applies primitive substitution (with binding $\lambda X \cdot \; X = a$ for *Q*)^5^ to derive

$$\begin{aligned}{}[a = a]^{\mathrm {f}\mathrm {f}}\vee [a = b]^{\mathrm {t}\mathrm {t}}\vee [b = a]^{\mathrm {t}\mathrm {t}}\end{aligned}$$

Pre-unification is applied and introduces clause

$$\begin{aligned}{}[a = b]^{\mathrm {t}\mathrm {t}}\vee [b = a]^{\mathrm {t}\mathrm {t}}\end{aligned}$$

The clauses $[a = b]^{\mathrm {t}\mathrm {t}}\vee [b = a]^{\mathrm {t}\mathrm {t}}$ , $[q a]^{\mathrm {t}\mathrm {t}}\vee [a = b]^{\mathrm {f}\mathrm {f}}$ and $[q b]^{\mathrm {f}\mathrm {f}}\vee [a = b]^{\mathrm {f}\mathrm {f}}$ (amongst others) have been identified by Leo-II as input candidates for a FO prover, and suitably converted copies of these clauses have been put into the FO store. In the next periodic call of a FO ATP (e.g. E) to this store, a refutation based on these three clauses is found and reported. Leo-II then stops its proof search, and, controlled by its flag settings, may even report a merged proof consisting of Leo-II’s and E’s contributions.

The above proof is obtained when using the simple (and older) fully-typed translation to FO logic (flag --translation fully-typed) and when the automated detection and replacement of Leibniz equations by primitive equations is disabled (flag --notReplLeibnizEQ). The current version of Leo-II employs more sophisticated translations to FO logic by default, as well as detecting Leibniz equations. Hence, in its latest default setting a shorter proof is obtained for SEV288ˆ5. In this proof clause $[(\forall Q \cdot \lnot Q a \vee Q b) = (a = b)]^{\mathrm {f}\mathrm {f}}$ is already converted into a refutable set of FO clauses for E.

Problem SEV288ˆ5 may be modified to obtain a slightly more challenging example. The outermost primitive equality may be replaced by a Leibniz equation to obtain

$$\begin{aligned} \forall R \cdot \; R (\lambda X_\alpha \lambda Y \forall Q \cdot \; Q X \longrightarrow Q Y) \longrightarrow R (\lambda X \lambda Y \cdot \; X = Y) \end{aligned}$$

The initialisation process and clause normalisation in Leo-II turns this problem into the following two clauses (where *r* is a fresh Skolem constant)

$$\begin{aligned}{}[r (\lambda X_\alpha \lambda Y \forall Q \cdot \; \lnot Q X \vee Q Y)]^{\mathrm {t}\mathrm {t}}\qquad [r (\lambda X \lambda Y \cdot \; X = Y)]^{\mathrm {f}\mathrm {f}}\end{aligned}$$

Leo-II resolves these two clauses together to obtain the pre-unification problem

$$\begin{aligned}{}[r (\lambda X_\alpha \lambda Y \forall Q \cdot \; \lnot Q X \vee Q Y) = r (\lambda X \lambda Y \cdot \; X = Y)]^{\mathrm {f}\mathrm {f}}\end{aligned}$$

After decomposing head symbol *r* the prover arrives at the situation as discussed above.

Like many other ATPs Leo-II has many flags which influence its detailed proof search behaviour [19]. Depending on their particular choice the prover may perform quite differently.

## Term Sharing and Term Indexing in Leo-II

Term indexing techniques are widely used in major FO ATPs [51, 52, 66]. The indexing data structures store large numbers of terms and, for a given query term *t*, support the fast retrieval of terms from the index that satisfy a certain relation with *t*. Examples of such relations include matching, unifiability, and syntactic equality [46]. Performance can be further enhanced by representing terms in efficient data structures, such as shared terms—these are used in E [52].

HO term indexing techniques are rarely addressed in the literature, which hampers the progress of systems in this field. An exception is Pientka [50]. Leo-II’s implementation at term level is based on a perfectly shared term graph, i.e., syntactically equal terms are represented by a single instance. Ideas from FO term sharing are adapted to HO logic by (1) keeping indexed terms in $\beta \eta$-normal form (i.e., $\eta$-short and $\beta$-normal) and (2) using de Bruijn indices [31] to allow $\lambda$-abstracted terms to be shared. The resulting data structure represents terms in a directed acyclic graph (DAG). Leo-II also supports the visualization of such term graphs^6^ and, more importantly, their statistical analysis. Future work will investigate whether such information can be exploited for improving heuristic control.

Representation of terms in a shared graph naturally advances the performance of a number of operations. For example, it allows fast lookup of all occurrences of syntactically equal terms or subterms, and it improves the performance of rewrite operations, such as global unfolding of definitions. Additionally, Leo-II employs a term-indexing data structure, which is based on structural indexing methods from the FO domain [42, 57], as well as road-sign techniques. Road signs are features of the data structure which guide operations based on graph traversal. They help to cut branches of the subgraph to be processed early and they are employed, e.g., in the construction of partial syntax trees [65] in which all branches with no occurrences of a given symbol or subterm are cut. This enables Leo-II to avoid potentially costly operations, such as occurs checks, and to speed up basic operations on terms, such as substitution.

## TPTP THF0 and Semantic Embeddings

Leo-II’s native input language is TPTP THF0 [64]. Particularly during 2008 and 2009, there has been a close collaboration and mutual fertilization between both evolving projects, and Leo-II and TPTP THF0 have been applied as mutual $\alpha$ testers. Fostered by the evolution of the TPTP THF infrastructure, HO ATP has recently made significant progress. At present there are at least six THF0-compliant provers and model finders available. These systems can be assessed online via the SystemOnTPTP tool [60], through which they can be easily employed avoiding local installations.

The recent progress in automating HO logic is measurable in terms of improvement rates in the yearly THF0 CASC competitions: 7 In 2010 the winner Leo-II performed 56 % better than the 2009 champion TPS, the 2011 winner Satallax was 21 % better than the 2010 champion Leo-II, in 2012 Isabelle was 10 % better than 2011 winner Satallax, and in 2013 winner Satallax-MaLeS was 21 % better than 2012 winner Isabelle.

**Fig. 2:**
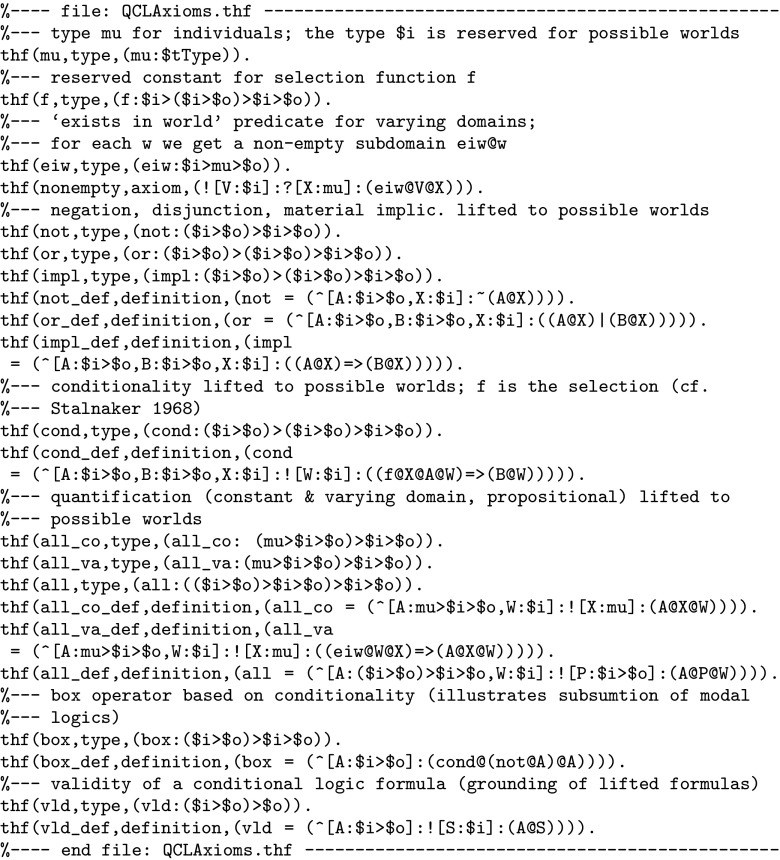
Example THF0 encoding of quantified conditional logics (QCLs). Kripke style semantics of QCL [56] is explicitly expressed in THF0. Varying and constant domain quantification are supported simultaneously. This embedding turns Leo-II (and any other THF0-compliant prover) into an reasoner for QCL

**Fig. 3:**
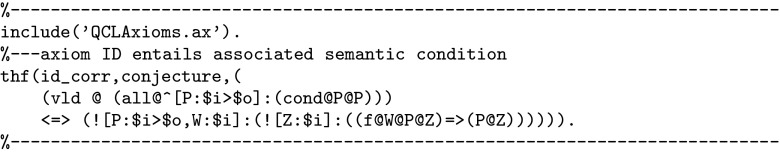
THF0 encoding of a well known correspondence between QCL axiom ID and a semantic condition of the selection function *f*

To illustrate THF0 syntax we present in Fig. 2 a small example theory. This example theory serves a second purpose for this article; namely it illustrates that quantified non-classical logics can be modeled as natural fragments of classical HO logic and that they can be be automated with provers like Leo-II. The particular logic embedded here is QCL—quantified conditional logic [56]. Benzmüller [11] presents the theory and more details on this embedding. The interesting point for this article is that these few axioms turn Leo-II (and any other THF0-compliant ATP) into a sound and complete reasoner for QCL. Note that even flexible combinations of varying and constant domain quantification are supported here. The family of QCLs have many applications, including AI and computational linguistics. They are challenging to automate and no other implemented provers for this logic currently exist. QCLs are very expressive and they e.g. subsume quantified modal logic (cf. the definition of box).

We now briefly describe THF0 syntax to explain the contents of Fig. 2. For details we refer to [64]. The symbols $i and $o represent the HO logic base types *i* (individuals) and *o* (propositions). The string $i>$o denotes the type of a function (more precisely, a predicate). Function or predicate application, for example, the proposition $$(eiw\,V\,X)$$, is encoded as ((eiw@V)@X) or simply as (eiw@V@X)—i.e., function application is represented by @, and it is left-associative. Taking $$\lambda {A}_{i\rightarrow o} \forall {S_i} (A\,S)$$ as an example expression, universal quantification and $$\lambda $$-abstraction are THF0-encoded as ˆ[A:$i>$o]:![S:$i]:(A@S). The symbol ? denotes the existential quantifier, and $$\lnot ,\vee ,\wedge ,\mathrm { and }\rightarrow $$ (material implication) are written as ~, |, &, and => . Comments begin with %. Better-formatted and more readable presentations of our THF0 code can easily be generated with the TPTP tools of [62]; here we optimised for less space.

Figure 3 formulates a well-known meta-level correspondence theorem for QCL: the axiom ID: $\forall P (P \Rightarrow P)$, where $\Rightarrow$ is the *conditional operator* (not be confused with material implication $\rightarrow$), is equivalent to the semantic condition $\forall P\forall w\forall z (f \, w \,P \, z \rightarrow P \, z)$ on the selection function *f* (the conditional operator $\Rightarrow$ appears as cond in Fig. 2). The statement in Fig. 3 can be proved in a few milliseconds by Leo-II. Benzmüller [11] presents prominent default reasoning examples from the AI literature that have been automated with this approach.

The Leo-II project has been active in submitting proof problems to the THF library. In particular, many examples in the spirit of Figs. 2 and 3, which illustrate the immediate applicability of THF0 reasoners for a wide range of non-classical logics, stem from the Leo-II initiative.

## Leo-II’s Proof Certificates

Running Leo-II on a problem can have several outcomes: the conjecture could be found to be a theorem, or found to be a non-theorem, or the prover could give up (because of a timeout, for instance). Leo-II conforms to the SZS standard ontology [61] for communicating the outcome of a proof attempt. This makes it easier for external tools to interpret this outcome.

In addition to this, Leo-II can also output a proof certificate. This details the justification for the outcome given by Leo-II, by providing the reasoning steps used by Leo-II to derive a refutation. This could then be used by an independent system to check Leo-II’s reasoning, or to use that derivation in a bigger formalisation. In Sect. 8 we describe how such certificates are imported into Isabelle/HOL, thus allowing us to translate Leo-II theorems into Isabelle/HOL theorems.

Leo-II can generate proof certificates in two levels of detail. When called with the option -po 1, Leo-II produces a proof containing the reasoning steps made by Leo-II alone—information on the reasoning made by the cooperating FO ATP are omitted. When called with option -po 2, Leo-II tries to merge the proof steps of the cooperating FO ATP with its own steps in order to return a joint THF-FOF proof object [59]. The -po 2 mode is unfortunately still very brittle and therefore not yet recommended for extensive use.

Leo-II’s proof certificates are encoded in the TPTP TSTP syntax [63], in which each inference is encoded as an annotated formula. The inference’s conclusion appears as the formula (e.g., in THF0 or FOF syntax), and the inference’s hypotheses and other meta-data are referenced or encoded in the formula’s annotations. Examples of proofs in both levels of detail are provided on the Leo-II website, at http://christoph-benzmueller.de/leo/download.html.

## Importing Leo-II Proofs into Isabelle/HOL

Proof certificates produced by ATPs are usually not for human consumption. Unlike proofs in natural language, it is very difficult to extract an intuition from such machine-found proofs, and this makes them difficult to understand and check manually. This also applies to Leo-II’s HO resolution proofs.

Additional automated tools can be used to check such proofs. Leo-II’s proofs can be imported into the proof assistant Isabelle/HOL, and the import only succeeds if Isabelle/HOL succeeds in replaying Leo-II’s proof. Once a proof is imported, it can be used in other formal developments within Isabelle/HOL.

The reconstruction involves the following stages:

1. The TPTP proof is parsed, and the Isabelle/HOL signature is extended with the types and constants appearing in the TPTP proof. Then the formula comprising each inference is interpreted as an Isabelle/HOL formula.
2. The proof is represented as a directed acyclic graph: vertices consist of formulas, and arcs connect conclusions with hypotheses. Formulas are annotated with inference-related information, such as the name of the inference rule used by Leo-II to derive that formula.
3. Proofs often need to be transformed prior to reconstruction. Transformation serves to *simplify* the proof—for instance, it could remove redundant inferences, or break inferences down into simpler inferences—and to *analyse* the proof to obtain information that can help guide reconstruction—such as finding applications of splitting rules.
  At the end of this process a *proof skeleton* is obtained, which is encoded using a simple intermediate language. Expressions in this skeleton will be interpreted by a virtual machine at a later stage, to complete the reconstruction.
4. The set of inferences involved in a proof is extracted, using the graph from Step 2. Using the inference name for guidance, each Leo-II inference is interpreted as an Isabelle/HOL inference, using specialised tactics. The resulting Isabelle/HOL inferences are stored in a dictionary. Taken together, these tactics serve as a mechanical implementation of Leo-II’s calculus in Isabelle/HOL—excluding key features related to proof-search, such as the given-clause algorithm and related data structures. However, some limited proof-search capabilities have been implemented. This was intended to make the reconstruction more robust, and also to reconstruct compound inferences (from shorter proof scripts). The resulting implementation is a mini-prover that is parametrised by a set of rules: during proof search the prover only uses a rule if it is in that set.
5. Finally, the proof skeleton from Step 3, enriched with the dictionary of inferences from the previous step, is evaluated.

Leo-II’s reliance on collaboration with other provers complicates proof reconstruction since Leo-II’s proofs may be *hybrid* proofs (cf. Sect. 7), consisting of contributions from different provers. We currently only handle pure Leo-II proofs. Our approach is compositional, and should be able to handle hybrid proofs, but it remains to implement the FO ATP part.

The mapping of pure Leo-II proofs into Isabelle/HOL theorems is crucial to Step 4. Intuitively, starting from the fact that every Leo-II type and term is an Isabelle/HOL type or term, and then showing that every Leo-II inference can be emulated in Isabelle/HOL, we can show that any Leo-II proof can be interpreted as an Isabelle/HOL theorem. We tested the reconstructor on THF problems from TPTP v5.4.0, and were able to reconstruct over 93 % of the proofs found by Leo-II.

## Applications of Leo-II

Section 6 describes how QCLs can be modeled and automated as natural fragments of classical HO logic. In fact, many well-known non-classical logics can be analogously embedded in HO logic and automated with Leo-II. In recent years this approach has inter alia been studied for a range of quantified modal logics [18], security logics [9] and intuitionistic logic [17]. Moreover, classical HO logic is suited as a uniform framework for combining embedded logics [10, 12]. In all this research the Leo-II prover has been the primary debugging tool supporting the formalization process and initial experiments.

For many challenging logics, like QCLs or HO modal logics, no theorems provers in the direct approach have been implemented yet. By exploiting the embedding approach, Leo-II and Satallax have pioneered the automation of such expressive logics, which have many applications [23].

Leo-II played a key role in the formalization, mechanization and automation of Gödel’s ontological proof of the existence of God [21, 22]; the THF0 formalization and further information is available online at http://github.com/FormalTheology/GoedelGod/. The system was extensively used during the formalization, and it was the first prover to fully automate the four steps as described in the notes on Gödel’s proof by Dana Scott [54]. Leo-II’s result was subsequently confirmed by Satallax. Interestingly, Leo-II can prove that Gödel’s original axioms [55] are inconsistent: in these notes definition D2 (*An* essence * of an individual is a property possessed by it and necessarily implying any of its properties*: ) is lacking conjunct $\phi (x)$, which has been added by Scott. Gödel’s axioms are consistent only with this conjunct present. The guess of a suitable instantiation for a predicate (set) variable via primitive substitution is a key step in Leo-II’s inconsistency proof. Leo-II’s inconsistency result is new; it has not been reported in philosophy publications. Meanwhile Leo-II has have been successfully employed in further experiments in metaphysics [28].

Leo-II also performed well in experiments related to the Flyspeck project of Hales [35], in which a formalised proof of the Kepler conjecture has been developed (mainly) in HOL Light. In those experiments [41, Table 7], which inter alia investigated the potential of several ATPs for automating subgoals in the Flyspeck corpus, Leo-II performed better than many prominent FO provers, including Vampire, Satallax, and SPASS. On the other hand, the E-based Leo-II prover performed worse than E itself on this corpus. There are a number of possible reasons, including the different input encodings used in the experiments for HO and FO ATPs, and the fact that E serves in Leo-II only as a subordinate reasoner whose full potential for automating FO fragments of HO logic is still not optimally exploited.

It has also been shown that Leo-II can be employed for reasoning in expressive ontologies, when it was integrated with the Sigma ontology engineering tool [48]. In recent experiments [24], Leo-II was used to detect errors in the SUMO ontology that cannot be detected by FO ATPs when applied to SUMO [1, 49].

Leo-II has recently also been integrated with the heterogeneous tool set Hets [45].

## Conclusion and Future Work

The development of the standalone resolution-based HO ATP Leo-II had a strong influence on some relevant and important developments, most notably the development of TPTP THF0 (which, goaded by the yearly CASC competitions in the THF0 category, fostered significant overall progress in HO ATP), the automation of quantified non-classical logics with HO ATPs, and the integration of heterogeneous provers. The latter aspect is pursued in the Leo-II project in two ways: Leo-II internally cooperates with external ATPs, and it has itself been integrated with other systems (such as Isabelle/HOL) which can verify proofs produced by Leo-II.

There remains much room for future work, including, for example, the incorporation of term orderings in Leo-II’s proof calculus and proof search, the integration of integer arithmetic, polymorphism and a calculus level support for induction.

## Footnotes

1 Cf. TPTP problem files SET171+3.p and SET171ˆ3.p and their solutions. These files contain encodings of the distributive law for union and intersection. In a way, the FO encoding provided in the former file is already tailored to simplify the proof search, since irrelevant but challenging set theory axioms are omitted. This is not the case for the latter HO encoding. SET171ˆ3.p can be solved by Leo-II and Satallax in a few milliseconds, while prominent FO provers like Vampire (version 3.0), E (version 1.9) and SPASS (version 3.7) time out (after 300 seconds) for SET171+3.p according to the information provided on TPTP.

2 Andrews [3, p. 207] gives the Comprehension Axiom scheme as $\exists U^{\sigma \rightarrow \tau } \forall V^\sigma \cdot U V = \mathbf {A}^\tau$ which, when written in $\lambda$-notation, shows up as the $\beta$-conversion rule.

3 The pre-unification depth is a parameter in Leo-II that can be specified at the command line. By default Leo-II currently operates with values up to depth 8. So far there has been no exhaustive empirical investigation of the optimal setting of the pre-unification depth.

4
Leo-II employs some constraints regarding the Boolean extensionality rule. In particular, application of the rule is enabled only when unification constraint $[\mathbf {M}^o = \mathbf {N}^o]^{\mathrm {f}\mathrm {f}}$ is the result of another pre-unification rule such as decomposition, that is, entry level applications of the Boolean extensionality rule are not permitted.

5 A more general approximate binding is $\lambda X \cdot \; (R X) = (S X)$, where *R* and *S* are fresh variables; however, Leo-II supports additional, heuristically motivated bindings such as the one used here.

6 To further visualise the evolution of the term graph during proof search, Leo-II has been modified to output a snapshot of its state after each processing step. This data was used to create animations of dynamically changing term graphs during proof search. The video clips can be obtained at http://christoph-benzmueller.de/leo/art.html.

7
http://www.cs.miami.edu/~tptp/CASC/.

## References

[1] Álvez J, Lucio P, Rigau G (2012). Adimen-SUMO: reengineering an ontology for first-order reasoning. Int. J. Semantic Web Inf. Syst..

[2] Andrews PB (1989). On connections and higher order logic. J. Autom. Reason..

[3] Andrews PB (2002). An Introduction to Mathematical Logic and Type Theory: To Truth Through Proof, Applied Logic Series.

[4] Andrews PB, Bishop M, Issar S, Nesmith D, Pfenning F, Xi H (1996). TPS: a theorem-proving system for classical type theory. J. Autom. Reason..

[5] Backes J, Brown CE (2011). Analytic tableaux for higher-order logic with choice. J. Autom. Reason..

[6] Benzmüller, C.: A calculus and a system architecture for extensional higher-order resolution. Research Report 97-198, Department of Mathematical Sciences, Carnegie Mellon University, Pittsburgh, USA (1997)

[7] Benzmüller, C.: Extensional higher-order paramodulation and RUE-resolution. In: Ganzinger, H. (ed.) Automated Deduction—CADE-16, 16th International Conference on Automated Deduction, Trento, Italy, July 7–10, 1999, Proceedings, no. 1632 in LNCS, pp. 399–413. Springer (1999). doi:10.1007/3-540-48660-7_39

[8] Benzmüller C (2002). Comparing approaches to resolution based higher-order theorem proving. Synthese.

[9] Benzmüller, C.: Automating access control logic in simple type theory with LEO-II. In: Gritzalis, D., López, J. (eds.) Emerging Challenges for Security, Privacy and Trust, 24th IFIP TC 11 International Information Security Conference, SEC 2009, Pafos, Cyprus, May 18–20, 2009. Proceedings, IFIP, vol. 297, pp. 387–398. Springer (2009). doi:10.1007/978-3-642-01244-0_34

[10] Benzmüller C (2011). Combining and automating classical and non-classical logics in classical higher-order logic. Ann. Math. Artif. Intell. (CLIMA XI).

[11] Benzmüller, C.: Automating quantified conditional logics in HOL. In: Rossi, F. (ed.) 23rd International Joint Conference on Artificial Intelligence (IJCAI-13), pp. 746–753. Beijing, China (2013a)

[12] Benzmüller, C.: A top-down approach to combining logics. In: Proceedings of the 5th International Conference on Agents and Artificial Intelligence (ICAART), pp. 346–351. SciTePress Digital Library, Barcelona (2013b). doi:10.5220/0004324803460351

[13] Benzmüller C, Delahaye D, Woltzenlogel Paleo B (2015). Higher-order automated theorem provers. All about Proofs, Proof for All, Mathematical Logic and Foundations.

[14] Benzmüller, C., Brown, C.: A structured set of higher-order problems. In: Hurd, J., Melham, T.F. (eds.) Theorem Proving in Higher Order Logics, 18th International Conference, TPHOLs 2005, Oxford, UK, August 22–25, 2005, Proceedings, Springer, no. 3603 in LNCS, pp. 66–81 (2005). doi:10.1007/11541868_5

[15] Benzmüller, C., Brown, C.: The curious inference of Boolos in MIZAR and OMEGA. In: Matuszewski, R., Zalewska, A. (eds.) From Insight to Proof - Festschrift in Honour of Andrzej Trybulec, Studies in Logic, Grammar, and Rhetoric, vol. 10(23), pp. 299–388. The University of Bialystok, Polen (2007)

[16] Benzmüller, C., Kohlhase, M.: LEO—a higher-order theorem prover. In: Kirchner, C., Kirchner, H. (eds), Automated Deduction—CADE-15, 15th International Conference on Automated Deduction, Lindau, Germany, July 5–10, 1998, Proceedings, Springer, no. 1421 in LNCS, pp. 139–143 (1998). doi:10.1007/BFb0054256

[17] Benzmüller C, Paulson L (2010). Multimodal and intuitionistic logics in simple type theory. Logic J. IGPL.

[18] Benzmüller C, Paulson L (2013). Quantified multimodal logics in simple type theory. Logica Universalis.

[19] Benzmüller, C., Sultana, N.: LEO-II version 1.5. In: Blanchette, J.C., Urban, J. (eds.) PxTP 2013, EasyChair, EPiC Series, vol. 14, pp. 2–10 (2013)

[20] Benzmüller, C., Sultana, N.: Update report: LEO-II version 1.5. CoRR abs/1303.3761 (2013)

[21] Benzmüller, C., Woltzenlogel Paleo, B.: Formalization, mechanization and automation of Gödel’s proof of God’s existence (2013). arXiv:1308.4526

[22] Benzmüller, C., Woltzenlogel Paleo, B.: Automating Gödel’s ontological proof of God’s existence with higher-order automated theorem provers. In: Schaub, T., Friedrich, G., O’Sullivan, B. (eds.) ECAI 2014, IOS Press, Frontiers in Artificial Intelligence and Applications, vol. 263, pp. 93–98 (2014). doi:10.3233/978-1-61499-419-0-93

[23] Benzmüller, C., Woltzenlogel Paleo, B.: Higher-order modal logics: Automation and applications. In: Paschke, A., Faber, W. (eds.) Reasoning Web 2015, no. 9203 in LNCS, pp. 1–43. Springer, Berlin (2015). doi:10.1007/978-3-319-21768-0_2

[24] Benzmüller, C., Ziener, M.: Automated consistency checking of expressive ontologies—beware of the wrong interpretation of success!. In: Fink, M., Homola, M., Mileo, A., Varzinczak, I.J. (eds.) The 5th International Workshop on Acquisition, Representation and Reasoning with Contextualized Knowledge (ARCOE-LogIC 2013). Corunna, Spain (2013)

[25] Benzmüller C, Brown C, Kohlhase M (2004). Higher-order semantics and extensionality. J. Symb. Log..

[26] Benzmüller C, Sorge V, Jamnik M, Kerber M (2008). Combined reasoning by automated cooperation. J. Appl. Log..

[27] Benzmüller C, Brown C, Kohlhase M (2009). Cut-simulation and impredicativity. Log. Methods Comput. Sci..

[28] Benzmüller C, Weber L, Woltzenlogel Paleo B, Silvestre RS, Béziau JY (2015). Computer-assisted analysis of the Anderson-Hájek ontological controversy. Handbook of the 1st World Congress on Logic and Religion.

[29] Blanchette, J.C., Böhme, S., Popescu, A., Smallbone, N.: Encoding monomorphic and polymorphic types. In: Piterman, N., Smolka, S.A. (eds.) Proceedings of TACAS 2013, LNCS, vol. 7795, pp. 493–507. Springer (2013). doi:10.1007/978-3-642-36742-7_34

[30] Brown, C.: Satallax: an automatic higher-order prover. In: Gramlich, B., Miller, D., Sattler, U. (eds.) Automated Reasoning (IJCAR 2012), LNCS, vol. 7364, pp. 111–117. Springer, Berlin (2012). doi:10.1007/978-3-642-31365-3_11

[31] de Bruijn N (1972). Lambda-calculus notation with nameless dummies: a tool for automatic formula manipulation with application to the Church-Rosser theorem. Indag. Math..

[32] Claessen, K., Lillieström, A., Smallbone, N.: Sort it out with monotonicity. In: Proceedings of CADE-23, LNAI, vol. 6803, pp. 207–221. Springer (2011)

[33] Digricoli VJ, Harrison MC (1986). Equality-based binary resolution. J ACM.

[34] Gordon M, Melham T (1993). Introduction to HOL: A Theorem-Proving Environment for Higher-Order Logic.

[35] Hales, T: Mathematics in the Age of the Turing Machine. ArXiv e-prints arXiv:1302.2898 (2013)

[36] Harrison, J.: HOL Light: An overview. In: Proceedings of TPHOLs 2009, LNCS, vol. 5674, pp. 60–66. Springer (2009)

[37] Huet, G.: A complete mechanization of type theory. In: Proceedings of the 3rd International Joint Conference on Artificial Intelligence , pp. 139–146 (1973a)

[38] Huet G (1975). A unification algorithm for typed lambda-calculus. Theor. Comput. Sci..

[39] Huet, G.P.: Constrained resolution: a complete method for higher order logic. Ph.D. thesis, Case Western Reserve University (1972)

[40] Huet, G.P.: A mechanization of type theory. In: Proceedings of the 3rd International Joint Conference on Artificial Intelligence, pp. 139–146 (1973b)

[41] Kaliszyk C, Urban J (2014). Learning-assisted automated reasoning with flyspeck. J. Autom. Reason..

[42] McCune W (1992). Experiments with discrimination-tree indexing and path indexing for term retrieval. J. Autom. Reason..

[43] Miller, D.: Proofs in higher-order logic. Ph.D. thesis, Carnegie Mellon University (1983)

[44] Mints G (1999). Cut-elimination for simple type theory with an axiom of choice. J. Symb. Log..

[45] Mossakowski, T., Maeder, C., Lüttich, K.: The heterogeneous tool set, Hets. In: Proceedings of TACAS 2007, LNCS, vol. 4424, pp. 519–522. Springer (2007)

[46] Nieuwenhuis, R., Hillenbrand, T., Riazanov, A., Voronkov, A.: On the evaluation of indexing techniques for theorem proving. In: Proceedings of IJCAR-01, LNAI, vol. 2083, pp. 257–271. Springer (2001)

[47] Nipkow, T., Paulson, L., Wenzel, M.: Isabelle/HOL: A Proof Assistant for Higher-Order Logic. No. 2283 in LNCS. Springer (2002)

[48] Pease A, Benzmüller C (2013). Sigma: an integrated development environment for formal ontology. AI Commun..

[49] Pease, A., Sutcliffe, G.: First order reasoning on a large ontology. In: Urban, J., Sutcliffe, G., Schulz, S. (eds.) Proceedings of the CADE-21 Workshop on Empirically Successful Automated Reasoning in Large Theories, no. 257 in CEUR Workshop Proceedings, pp. 59–69 (2007)

[50] Pientka, B.: Higher-order substitution tree indexing. In: Palamidessi, C. (ed.) Proceedings of ICLP 2003, LNCS, vol. 2916, pp. 377–391. Springer (2003)

[51] Riazanov A, Voronkov A (2002). The design and implementation of VAMPIRE. AI Commun..

[52] Schulz S (2002). E - A brainiac theorem prover. AI Commun..

[53] Siekmann J, Benzmüller C, Autexier S (2006). Computer supported mathematics with OMEGA. J. Appl. Log..

[54] Sobel, J.: Logic and Theism: Arguments for and Against Beliefs in God, Cambridge U. Press, chap Appendix B. Notes in Dana Scott’s Hand, pp. 145–146 (2004a)

[55] Sobel, J.: Logic and Theism: Arguments for and Against Beliefs in God, Cambridge U. Press, chap Appendix A. Notes in Kurt Gödel’s Hand, pp. 144–145 (2004b)

[56] Stalnaker, R.: A theory of conditionals. In: Studies in Logical Theory, Oxford, pp. 98–112 (1968)

[57] Stickel, M.: The path-indexing method for indexing terms. Tech. Rep. 473, Artificial Intelligence Center, SRI International, 333 Ravenswood Ave., Menlo Park, CA 94025 (1989)

[58] Sultana, N.: Higher-order proof translation. Ph.D. thesis, Computer Laboratory, University of Cambridge, Available as Tech Report UCAM-CL-TR-867 (2015)

[59] Sultana, N., Benzmüller, C.: Understanding LEO-II’s proofs. In: Korovin, K., Schulz, S., Ternovska, E. (eds.) IWIL 2012, EasyChair, Merida, Venezuela, EPiC Series, vol. 22, pp. 33–52 (2013)

[60] Sutcliffe, G.: TPTP, TSTP, CASC, etc. In: Diekert, V., Volkov, M., Voronkov, A. (eds.) Proceedings of the 2nd International Computer Science Symposium in Russia, pp. 7–23. Springer, LNCS (2007)

[61] Sutcliffe, G.: The SZS ontologies for automated reasoning software. In: LPAR Workshops, CEUR Workshop Proceedings (http://ceur-ws.org/), vol. 418 (2008)

[62] Sutcliffe G (2009). The TPTP problem library and associated infrastructure. J. Autom. Reason..

[63] Sutcliffe, G.: The TPTP World—Infrastructure for Automated Reasoning. In: Proceedings of LPAR-16, no. 6355 in LNAI, pp. 1–12. Springer (2010)

[64] Sutcliffe G, Benzmüller C (2010). Automated reasoning in higher-order logic using the TPTP THF infrastructure. J. Formaliz. Reason..

[65] Theiß, F., Benzmüller, C.: Term indexing for the LEO-II prover. In: IWIL-6 workshop at LPAR, : The 6th International Workshop on the Implementation of Logics. Pnom Penh, Cambodia (2006)

[66] Weidenbach, C., Brahm, U., Hillenbrand, T., Keen, E., Theobald, C., Topic, D.: Spass version 2.0. In: Voronkov, A. (ed) Proceedings of CADE 2002, LNCS, vol. 2392, pp. 275–279. Springer (2002)

